# Numerical Simulations for Damage and Failure of a Polymer Material Subjected to Thermal Fatigue Loading

**DOI:** 10.3390/polym17091153

**Published:** 2025-04-23

**Authors:** Jun Koyanagi, Takumu Sugiyama, M. J. Mohammad Fikry, Yutong Li, Takuhei Tsukada

**Affiliations:** 1 Department of Materials Science and Technology, Faculty of Advanced Engineering, Tokyo University of Science, 6-3-1 Niijuku, Katsushika-ku, Tokyo 125-8585, Japan; liyutong@rs.tus.ac.jp; 2 Department of Materials Science and Technology, Graduate School of Advanced Engineering, Tokyo University of Science, 6-3-1 Niijuku, Katsushika-ku, Tokyo 125-8585, Japan; 8224536@ed.tus.ac.jp; 3 Department of Mechanical Engineering, College of Engineering and Polymer Science, The University of Akron, 244 Sumner St., Akron, OH 44325-3903, USA; mfikry@uakron.edu; 4 Polyplastics Co., Ltd., 973 Miyajima, Fuji, Shizuoka 416-8533, Japan; takuhei.tsukada@polyplastics.com

**Keywords:** thermal fatigue, numerical simulation, entropy damage criterion, finite element analysis

## Abstract

This study proposes a novel numerical approach to simulate damage accumulation and failure in polymer materials under thermal fatigue, using an entropy-based damage criterion. Unlike the many experimental studies in this area, few numerical simulations exist due to the complexity of modeling thermal fatigue. In our method, thermal and mechanical stresses arising from thermal expansion mismatches and temperature gradients are modeled through a coupled simulation approach. A viscoelastic constitutive equation is implemented in ABAQUS via a user-defined subroutine to capture damage progression. The method includes surface and internal thermal conduction, thermal deformation, and time–temperature superposition using reduced viscosity, enabling accurate simulation under varying thermal conditions. The results show that localized thermal stresses induced by temperature gradients lead to progressive damage and failure. This study demonstrates the first successful numerical simulation of thermal fatigue-induced damage in polymer materials. The proposed framework reduces the need for extensive experiments and offers insights into residual stress prediction and durability evaluation, contributing to polymer design and application in high-performance environments.

## 1. Introduction

The damage and failure of composite materials consisting of reinforcement and polymer matrix subjected to thermal fatigue loading constitutes a pressing problem in modern engineering. Extensive research has been conducted to examine thermal fatigue damage, primarily through experimental approaches. Early studies by Biernacki et al. [1] and Shin et al. [2] provided foundational insights into the mechanical behavior of composites under cyclic thermal loading, focusing on large-scale experimental studies and failure predictions in graphite/epoxy composites exposed to simulated space conditions. Kobayashi et al. [3,4] expanded on this by investigating damage mechanics in carbon fiber-reinforced plastic (CFRP) laminates, emphasizing matrix cracking and the long-term durability of high-temperature-resistant composites. Meanwhile, Smmazçelik et al. [5] explored how thermal cycling affects impact fatigue properties in thermoplastic matrix composites, demonstrating the performance degradation that occurs under repeated thermal exposure. More recently, Deng et al. [6] and Lizarralde et al. [7] examined thermal aging effects and damage evolution in polymer-based composites, leveraging advanced imaging techniques such as X-ray computed tomography to assess internal structural changes over time.

While experimental studies have provided valuable insights, a deeper understanding of the underlying damage mechanisms remains critical. One of the most widely studied failure modes in thermally cycled composites is microcracking. Zrida et al. [8] and Ajaja and Barthelat [9] highlighted how microcracks develop in carbon fiber/polyimide laminates, progressively compromising their mechanical integrity. To further investigate this degradation process, Zhang et al. [10] employed numerical modeling, linking microcrack propagation to overall mechanical deterioration in triaxial braided composites. Additionally, Qiu et al. [11] examined the mechanical behavior of carbon fiber/epoxy laminates under thermal cycling, revealing reductions in stiffness and residual strength. Boccaccini et al. [12] also contributed to this area by studying Nicalon™-fiber-reinforced glass–matrix composites, demonstrating how fiber architecture influences thermal fatigue resistance. More recently, microscopic damage accumulation in specialized composite structures has been explored, particularly in aerospace and civil infrastructure applications. Kobayashi et al. [13] investigated fiber–matrix interactions in CFRP laminates used in high-precision satellite antennas, providing critical insights into micro-damage progression. Meanwhile, Mosallam et al. [14] examined the residual mechanical properties of triaxial CFRP laminates subjected to both thermal cycling and ultraviolet radiation, emphasizing their durability in bridge structures.

Beyond understanding failure mechanisms, researchers have also examined how thermal cycling impacts the mechanical properties of composite materials. Park et al. [15] and Shivakumar and Holloway [16] studied unidirectional carbon fiber/epoxy composites subjected to vacuum thermal cycling, demonstrating their vulnerability under extreme temperature conditions, which is particularly relevant for aerospace applications. Hancox [17] provided a broader perspective on how thermal cycling affects polymer matrix composites, laying the groundwork for further investigations into structural stability. Meanwhile, Qin et al. [18] focused on adhesively bonded composite structures, showing that interfacial degradation in CFRP/aluminum alloy joints leads to a decline in bond strength over time. The role of material composition in thermal fatigue resistance has also been widely studied. Azimpour-Shishevan et al. [19] investigated the effects of thermal cycling on carbon fiber-reinforced and basalt fiber-reinforced epoxy composites, highlighting the importance of polymer matrix selection in mitigating fatigue damage.

Of the many studies on thermal cycling damage in composites, nearly all have employed experimental approaches. Numerical simulation studies of damage accumulation under thermal fatigue are significantly limited, largely due to the complexity involved. It is worth noting that thermal fatigue failure in polymer composites is predominantly governed by the fatigue behavior of the polymer matrix. This is because the reinforcing fibers, such as carbon fibers, are almost unaffected under thermal cycling at temperatures around 400 K. Therefore, to truly understand and predict thermal fatigue damage in composites, it is essential to clarify the fatigue behavior of the polymer itself. Although some studies have addressed resin behavior under thermal environments, research that focuses specifically on thermal cycling of polymer resins alone is still quite limited. Most of the existing works tend to deal with thermal aging effects or combine mechanical loading with thermal fatigue [20,21,22]. This may reflect the fact that thermal stress fatigue in composites is closely related to the behavior of the polymer matrix itself. 

At the beginning, the thermal cycles induce two types of stress in polymer materials: one from mismatched thermal expansion in heterogeneous materials, and another from temperature gradients within the specimen even for homogeneous materials. These stresses create evolving histories that drive progressive damage accumulation. The complexity of this phenomenon has hindered the development of numerical simulations thus far. The challenges in modeling arise because each variable, such as the specimen geometry or thermal fatigue temperature, requires extensive experimental adjustment. A robust numerical simulation, however, would allow for the computational examination of thermal fatigue damage accumulation, significantly reducing experimental burdens. Such simulations would be instrumental in broadening the applications of composite materials. Therefore, we strongly believe that establishing an effective modeling approach is essential for advancing technical capabilities in research.

Researchers have investigated the failure of polymer materials using the entropy damage criterion [23,24,25,26,27,28,29,30,31,32]. Before any mechanical load is applied, the molecular alignment in the material exhibits a certain degree of order. Upon loading, this alignment becomes partially disrupted, resulting in an increase in entropy. If the deformation is entirely elastic, the molecular alignment returns to its original state once the load has been removed. However, if the deformation includes inelastic components, the molecular alignment does not fully recover, leading to a permanent increase in entropy. This behavior has been confirmed in our previous work using molecular dynamics simulations, which showed that cyclic deformations lead to the progressive increase in entropy within a system [33,34]. In the entropy damage criterion, entropy is directly correlated with material damage, and failure occurs when the entropy reaches a critical value. Recently, a growing number of studies have employed entropy as an index for predicting material failure [35,36,37,38,39,40,41,42,43,44,45,46,47,48,49,50]. The present study represents the first attempt to numerically model damage accumulation and failure due to thermal fatigue, which constitutes the original contribution of this paper.

Entropy is defined as the value of the dissipated energy divided by the absolute temperature [36,37,38]. Therefore, if the dissipated energy and temperature histories are known, the entropy increase at any moment can be calculated. By implementing a specific inelastic constitutive equation, including viscoelastic characteristics, into commercial finite element analysis software via a user subroutine, damage accumulation and failure under various loadings can be readily simulated [23,28,30]. Coupled simulations, including surface and internal thermal conduction, and the structural analysis of thermal deformations enable the accurate modeling of damage accumulation and failure under thermal fatigue. Notably, when the temperature rises, time is accelerated based on the time–temperature superposition principle [51]. Considering this, we apply a method herein using reduced viscosity, adjusted by an acceleration factor for each element, allowing for the accurate simulation of the damage accumulation behavior, even with temperature variations across the model.

The present study aims to numerically investigate the damage accumulation and failure behavior of polymer materials under thermal fatigue conditions (300–400 K), based on an entropy damage criterion. To achieve this, we use a previously developed viscoelastic model to analyze the thermal cycles of polymer materials [23]. The first case simulates thermal cycle failure in a system where all boundaries are completely constrained and subjected to temperature changes. Since the dimensions do not change, the temperature changes are directly translated into thermal stress; this leads to constant stress relaxation, which increases the dissipated energy and entropy, ultimately leading to failure. The second case simulates thermal cycling where the boundaries are not fixed, allowing free deformation. Since the material is assumed to be homogeneous, it can expand freely; however, stresses still arise due to temperature gradients within the material, leading to failure under thermal cycling. These analytical techniques present potential and notable advantages in real-world product manufacturing, helping to assess the damage in components that appear intact after molding. As demonstrated in [51,52], modeling the fibers, resin, and interfaces separately enables a more comprehensive approach to addressing the thermal fatigue problems in composite materials. In this study, however, we focus solely on the polymer resin.

## 2. Governing Equations and Material Parameters Used in This Study

Finite element analysis (FEM) was performed herein using the principle of virtual work, expressed as in Equation (1):

(1) $$\int_{V}\sigma_{ij}{\delta \varepsilon }_{ij}dV-\int_{V}F_{i}{\delta u}_{i}dS=0,$$

where $\sigma_{ij}$ represents the stress, ${\delta \varepsilon }_{ij}$ is the virtual strain, $F_{i}$ denotes the external force, and ${\delta u}_{i}$ is the virtual displacement.

To simulate the temperature changes, the transfer of heat from the surroundings to the resin was described using Equation (2), while the heat conduction within the resin was modeled with Equation (3):

(2) $$Q=Ah\left(T_{el}-T_{atm}\right)$$

(3) $$c\rho \frac{\partial T}{\partial t}=\lambda \left(\frac{\partial^{2}T}{\partial x^{2}}+\frac{\partial^{2}T}{\partial y^{2}}+\frac{\partial^{2}T}{\partial z^{2}}\right),$$

where Q is the heat flux; A is the surface area of the boundary where heat transfer occurs; h is the heat transfer coefficient; $T_{el}$ and $T_{atm}$ are the element and surrounding temperatures, respectively; c is the specific heat; ρ is the density; and λ represents the thermal conductivity.

The time–temperature superposition principle was applied herein to simulate the acceleration of time with increasing temperature. This is achieved using the shift factor $\alpha^{TR}$, which is defined by the relationship in Equation (4) [51]:

(4) $$\log\alpha^{TR}=\frac{∆H}{2.303R}\left(\frac{1}{T}-\frac{1}{T_{R}}\right),$$

where ∆H is the activation energy, R is the universal gas constant, T is the temperature, and $T_{R}$ is the reference temperature.

**Figure 1:**
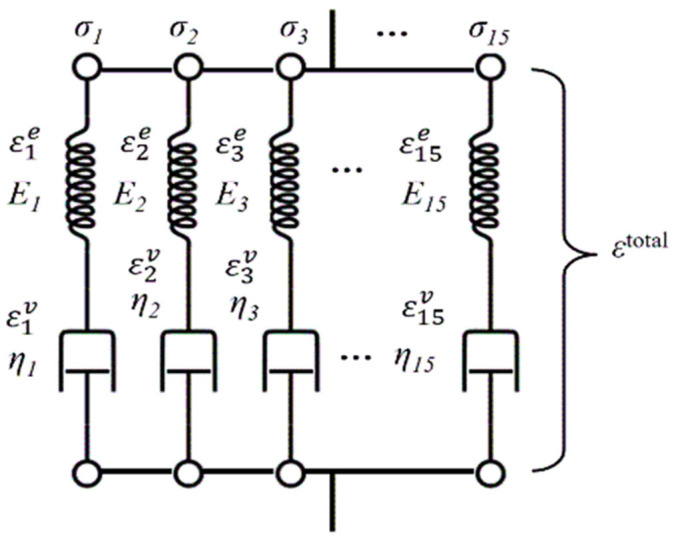
Numerical model for the constitutive equation.

The stress in the resin was calculated using a viscoelastic model to accurately represent the time-dependent mechanical behavior. The stress tensor was formulated as in Equation (5) [23]:

(5) $$\sigma_{ij}\left(t\right)=\left(1-D\right)\int_{0}^{t}E_{ijkl}^{r}\left(t-t^{\prime }\right)\frac{gd\varepsilon_{kl}^{ve}}{dt^{\prime }}dt^{\prime },$$

where $\varepsilon_{kl}^{ve}$ represents the viscoelastic strain, D is the damage variable, and $E^{r}$ is the relaxation modulus. $E^{r}$ was expressed as the summation of 15 Maxwell elements (Figure 1), as given by Equation (6):

(6) $$E^{r}(t)=\sum_{i=1}^{15}E_{i}\exp\left(-\frac{tE_{i}}{\eta_{i}}\right),$$

where $E_{i}$ is the elastic modulus, and $\eta_{i}$ is the viscosity coefficient of the i-th element. The nonlinear coefficient, g, was determined using Equation (7) [23]:

(7) $$g=\frac{1}{1+\alpha {\left(\frac{\sigma_{Mises}}{\sigma_{0}}\right)}^{m}},$$

where $\sigma_{Mises}$ is the von Mises stress, $\sigma_{0}$ is the reference stress, and α and m are material-specific constants obtained through experimental curve fitting. In this study, 15 Maxwell elements are employed in the viscoelastic constitutive model to accurately capture the complex time-dependent behavior of polymers under thermal fatigue loading. Compared to simplified models with fewer elements, this configuration provides higher resolution in representing the transient response during temperature and stress fluctuations. The number of elements was determined based on the authors’ previous work [23], where the same model was validated for cyclic loading conditions.

The dissipated energy during deformation was calculated using Equation (8) [30]:

(8) disE=σ·∆b,

where ∆b is the increment of dashpot deformation. The dissipated energy was then used to compute the entropy, as in Equation (9) [30]:

(9) $$W=\frac{disE}{T}\cdot \alpha_{d},$$

where T is the temperature, and $\alpha_{d}$ is a constant used to emphasize the effect of damage within the model. The damage variable D used in Equation (5) was formulated based on entropy accumulation, as in Equation (10) [30]:

(10) $$D=\left(W_{old}+\sum_{i=1}^{15}∆W\right)\times \frac{D_{cr}}{S_{cr}},$$

where $W_{old}$ represents the previously accumulated entropy, ∆W is the current increment in entropy, $S_{cr}$ is the critical entropy threshold, and $D_{cr}$ is the critical damage value.

**Figure 2:**
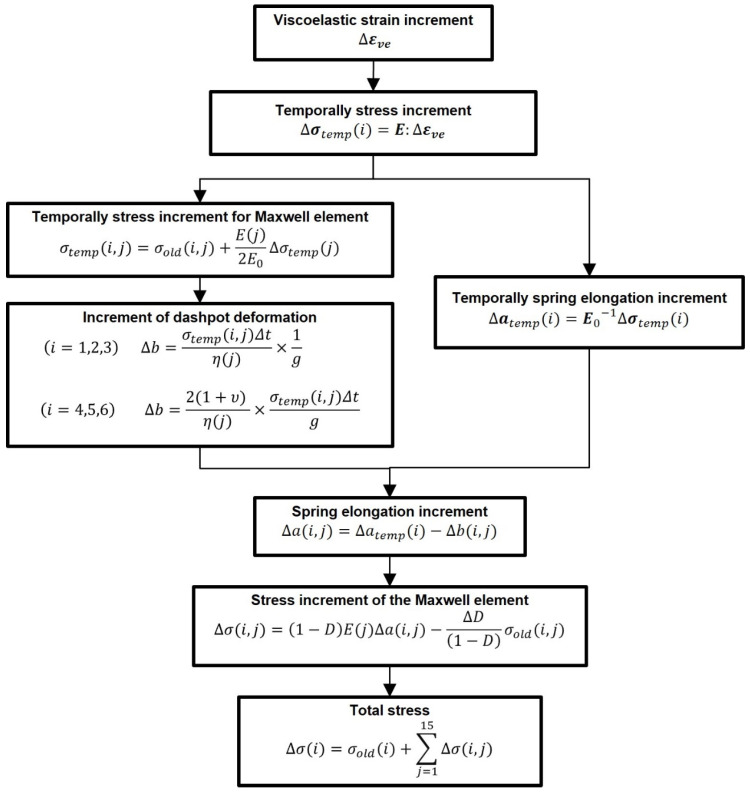
Flowchart of UMAT subroutine, including the viscoelastic behavior and entropy-based damage accumulation.

The abovementioned equations were implemented in a UMAT subroutine for the simulations in ABAQUS software (ABAQUS 2021/Standard). A flowchart of the UMAT subroutine is presented in Figure 2.

The material properties of the Maxwell elements, thermal parameters, entropy criterion, and damage variables used in the simulation are shown in Table 1. The parameters relating to viscoelastic characteristics are the same as in Ref. [23]. The parameters relating to thermal conduction are arbitrarily assumed to obtain variously different numerical results in the range of this study. The density was intentionally set to a lower value than the actual materials in order to accelerate the heat exchange process. This approach is equivalent to time-scaling and helps to simulate thermal fatigue behavior within a reasonable computational time.

*α_d_* is a factor of damage acceleration and the value is determined in order to represent various phenomena and enable us to qualitatively discuss the numerical results. The value is the same as in Ref. [30]. The value of *D_cr_* is also the same as in Ref. [30]. *S_cr_* is determined arbitrarily for the same purpose as mentioned above, and the values of *D_cr_* and *S_cr_* are individual. Consequently, the limitation of this numerical simulation is that the results do not exceed in the range of qualitative discussion. In this study, the viscoelastic constitutive model is assumed to be symmetric for tension and compression, and no distinction is made between the two. While this simplifies the analysis, further refinement will be needed to capture any asymmetry in compressive response.

## 3. Heat Cycle Failure Simulation with Fixed Displacement for Surface Nodes

In this section, the simulation conditions and results for the case of heat cycle failure under fixed displacement boundary conditions applied to surface nodes are presented and discussed.

### 3.1. Numerical Conditions

**Figure 3:**
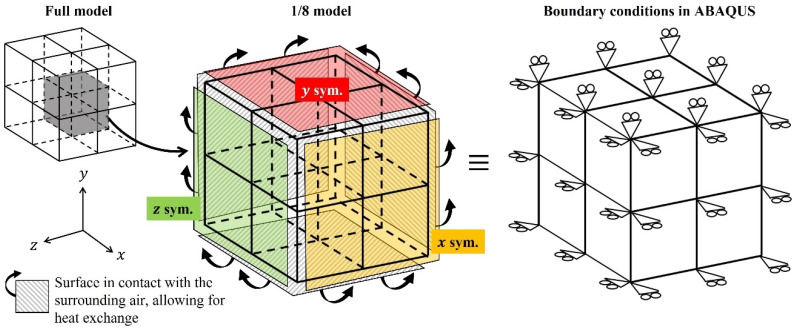
Numerical model and boundary conditions.

**Figure 4:**
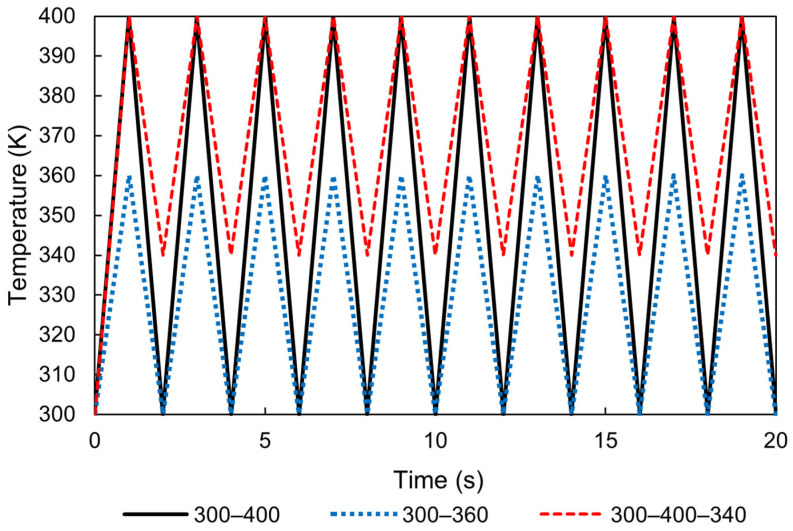
Input of atmospheric temperature for heat cycles, with ranges of 300–400 K, 300–360 K, and 340–400 K.

As shown in Figure 3, the analysis involved a 1/8 symmetrical model consisting of eight cubic elements, each with a side length of 1 mm. A coupled thermal–mechanical simulation approach was employed. Three of the faces were set as symmetry boundaries in the *x*-, *y*-, and *z*-directions, each intersecting with the origin, while the remaining three faces were constrained to keep the overall dimensions of the eight elements constant. This configuration effectively ensured that the total volume of the elements remained unchanged, simulating a scenario where the surrounding structure exhibits high rigidity with negligible thermal deformation. Heat transfer occurred exclusively through the three outer surfaces of the model, which were exposed to the surrounding air, enabling interactions and thermal exchange. The atmospheric temperature was set to follow a triangular waveform, fluctuating between 300 and 400 K, as illustrated in Figure 4.

### 3.2. Numerical Results

**Figure 5:**
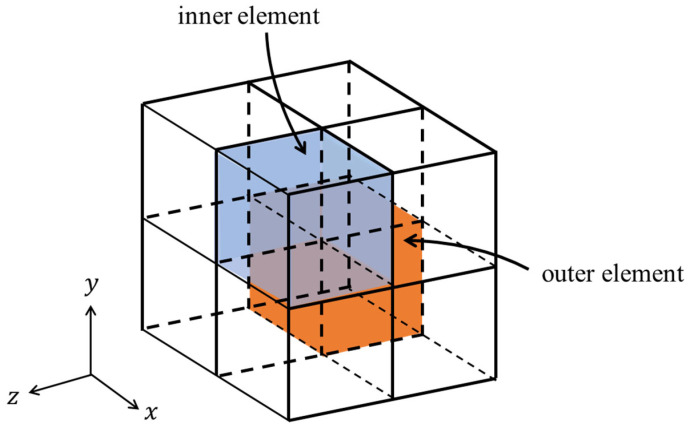
Schematic showing the locations of the elements analyzed in the study.

**Figure 6:**
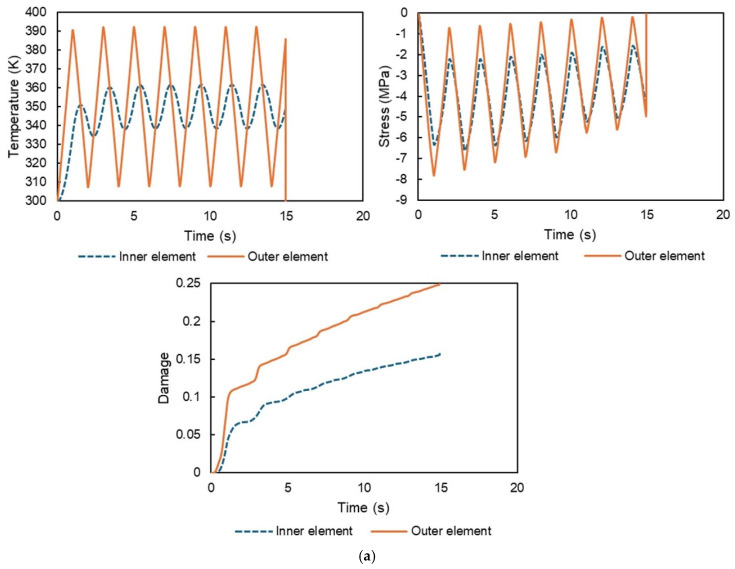

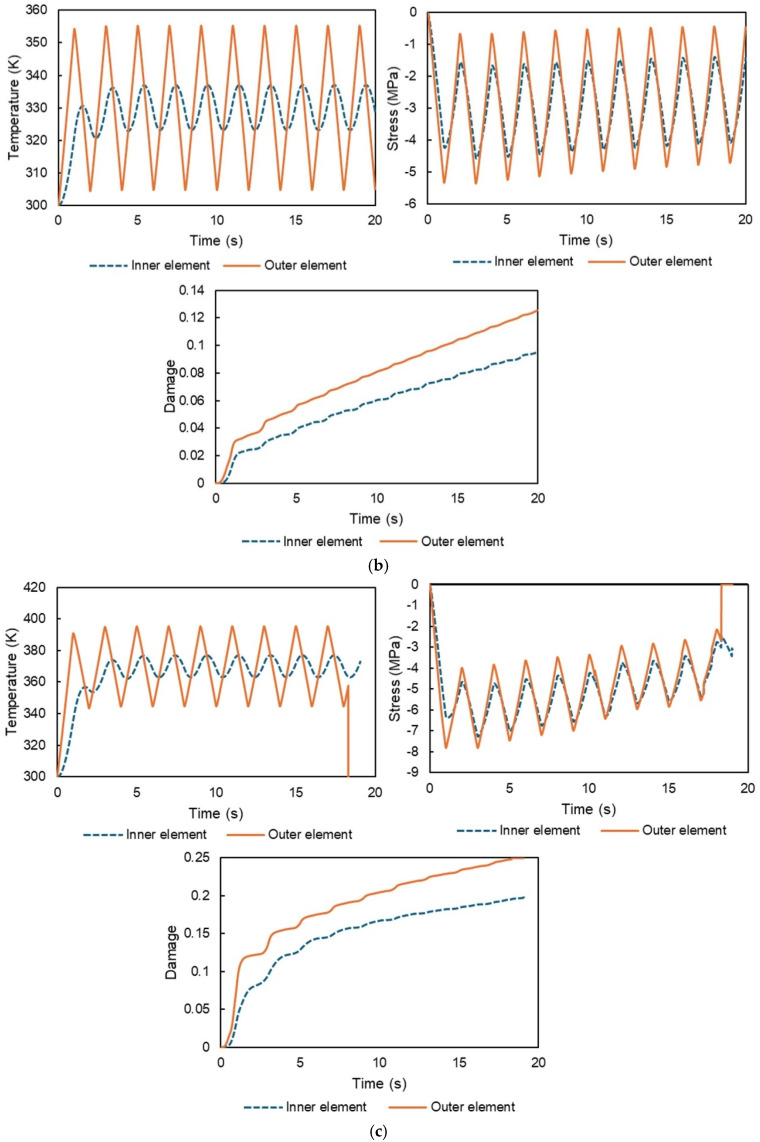
Temperature history, stress history, and damage progression of the inner and outer elements under different temperature ranges: (**a**) 300–400 K, (**b**) 300–360 K, and (**c**) 340–400 K.

As shown in Figure 5, elements with three surfaces exposed to the surrounding environment are referred to as outer elements, while those with no exposed surfaces are defined as inner elements. The temperature history, stress history, and resulting damage progression of these elements are presented in Figure 6 for different temperature ranges: (a) 300–400 K, (b) 300–360 K, and (c) 340–400 K. The temperature values were evaluated at the center of the elements, as were the stress values, specifically in the *z*-direction.

As shown in Figure 6, the temperature of the outer elements fluctuates significantly due to their exposure to the surrounding environment, whereas the temperature fluctuations of the inner elements remain less pronounced. This difference arises due to the time required for heat to propagate to the inner elements. Since the total volume of the model is fixed and only temperature changes are considered, the resulting thermal stress history is reasonable. As the deformation of the entire model is constrained, the outer elements exhibit larger axial forces compared to the inner elements, resulting in slightly higher absolute stress values for the former. We also confirmed that larger temperature variations lead to greater stress magnitudes. Furthermore, due to the viscoelastic nature of the material, the stress decreases over time. Even under the same stress conditions, different temperatures result in varying rates of damage accumulation. This behavior is attributed to the time–temperature superposition principle, which accelerates the time-dependent viscous behavior of the system. As a result, the dissipated energy increases, the entropy shows faster growth, and damage accumulates more rapidly. For instance, even with the same temperature range of 60 K, the case in Figure 6c exhibits faster damage progression than that in Figure 6b. Consequently, the elements with a greater number of surfaces exposed to the surrounding environment (outer elements) are the first to fail. Although the average temperature of the inner elements is almost identical to that of the outer elements, greater temperature fluctuations accelerate damage progression. In the case in Figure 6a, failure occurs after 15 s (one full thermal cycle corresponds to 2 s (heating and cooling), so the 15 s duration contains approximately 7.5 cycles); this accelerated failure is attributed to the material constants used in the study, which were intentionally selected to accelerate damage accumulation compared to the real components. In this study, damage was considered to correspond to failure after reaching the value of 0.25. In the range of 340–400 K, failure was observed at the 18th cycle, confirming that smaller temperature variations lead to a higher number of cycles before failure. This result aligns with our expectations and is considered reasonable.

The numerical simulations successfully capture the progressive accumulation of damage during thermal cycling. This analysis appears to be among the first to address this phenomenon, particularly in heterogeneous materials such as carbon fiber-reinforced plastic (CFRP); in such materials, the thermal expansion of the matrix is constrained by the fibers, similar to the boundary conditions employed in this analysis. Consequently, the results qualitatively demonstrate that resin damage accumulates during thermal cycling.

## 4. Heat Cycle Simulation with No Displacement Constraints

This section presents additional simulation results. The only change in the numerical conditions involved the removal of constraints on the surface nodes, allowing the model to deform freely with thermal expansion and contraction. This setup simulates typical thermal behavior in homogeneous materials, where thermal stress generally does not occur with temperature changes alone. However, in reality, temperature varies by position and changes dynamically over time with fluctuations in the ambient temperature. This temperature gradient induces localized, nonuniform thermal expansion, resulting in thermal stresses. Consequently, there is a risk of material failure due to ambient temperature changes. This phenomenon is similar to the rapid failure observed in large components immediately after fabrication when ambient cooling begins. Thermal stresses become more pronounced particularly in cases of rapid temperature changes, leading to potential material failure. This is similar to the fact that residual stresses can vary based on the history of ambient temperature exposure [53,54,55].

Herein, we simulated the damage accumulation and failure of viscoelastic materials subjected to cyclic ambient temperature changes. Similar to the previous simulations, the thermoviscoelastic behavior, thermal conduction, and damage accumulation were considered. The input ambient temperature, as shown in Figure 4, was applied again in this simulation. Further, symmetric boundary conditions were applied to the three faces, with no additional constraints.

**Figure 7:**
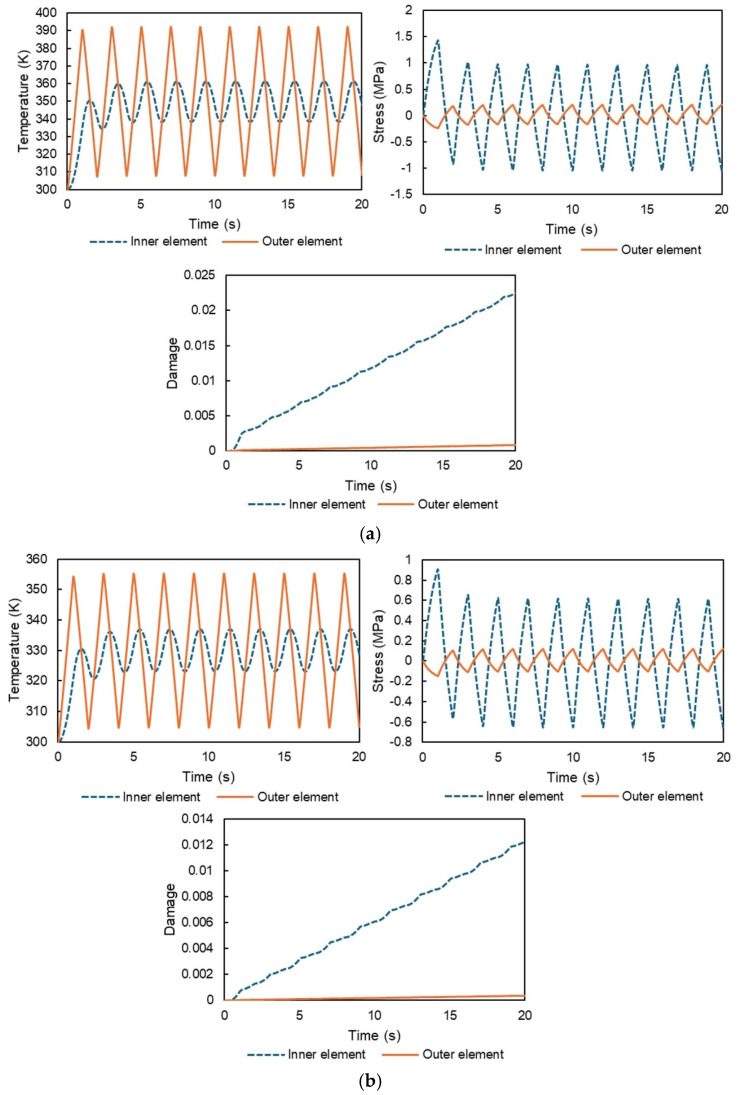

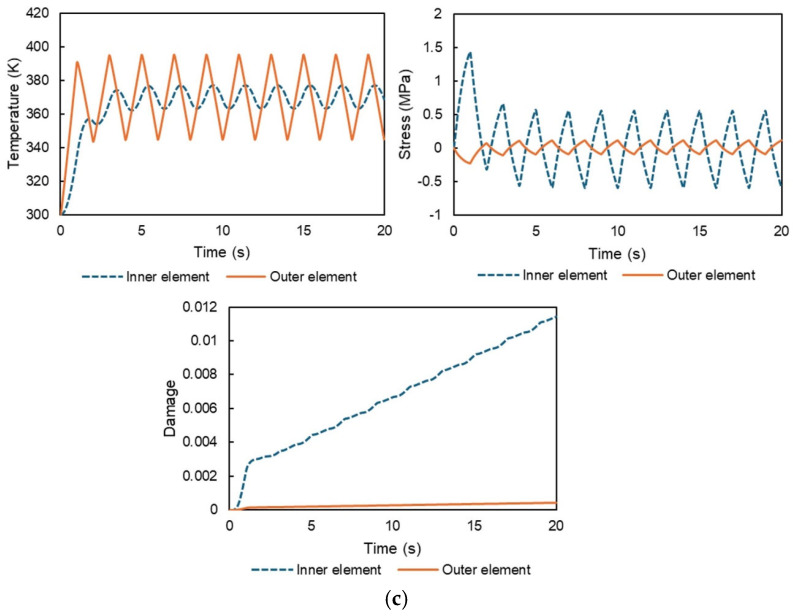
Temperature history, stress history, and damage progression of the inner and outer elements in the heat cycle simulation without displacement constraints under different temperature ranges: (**a**) 300–400 K, (**b**) 300–360 K, and (**c**) 340–400 K.

The results for the temperature history, stress history, and damage progression of the inner and outer elements under different temperature ranges—(a) 300–400 K, (b) 300–360 K, and (c) 340–400 K—are shown in Figure 7. As expected, the temperature of the outer nodes fluctuates more significantly due to the time lag in the heat transfer from the surface to the interior. The stress histories indicate relatively high stress near the center and lower stress near the surface; the stress arises from thermal expansion differences between the individual elements. The total stress must equal zero based on the stress equilibrium, which explains why the stress histories of the inner and outer regions are opposite in phase. Additionally, the inner elements experience a greater absolute stress than the surrounding elements including outer elements due to the smaller volume of the former.

Under the conditions of free thermal expansion, stress arises due to temperature gradients within the material. The inner elements experience higher stress levels compared to the external elements primarily because the regions exposed to larger temperature variations possess a greater volume. This disparity in stress levels suggests that the internal elements are more likely to fail earlier during thermal cycling. The analysis also reveals that the stresses in the inner and outer elements have opposite signs, consistent with the principles of force equilibrium. The larger absolute stress observed in the inner elements leads to faster damage progression in these regions. This phenomenon highlights that even in homogeneous materials, temperature differences within the structure can generate internal stresses during free thermal expansion, ultimately resulting in failure under cyclic thermal loading. This study is the first to successfully capture the temperature and stress histories required to numerically describe this behavior.

Although the current work is limited to qualitative simulations, the inclusion of thermoviscoelastic properties, realistic temperature profiles, and accurate material constants could enable quantitative predictions of damage evolution during thermal cycling. However, this relies on the applicability of the entropy-based damage criterion used in this study. The numerical method developed herein represents a significant advancement with the simulation of damage accumulation and failure under thermal cycling, a previously unexplored field. This approach is expected to contribute to various applications in the future, such as molding simulations and predictions of thermal residual stresses, offering valuable insights for industries that rely on high-performance materials.

## 5. Conclusions

This study developed a numerical simulation framework to investigate the damage accumulation and failure behavior of polymer materials subjected to thermal fatigue loading, based on an entropy-based damage criterion. A previously proposed viscoelastic model was implemented in finite element software to simulate thermal fatigue under two different boundary conditions: fixed displacement and free expansion. The simulations successfully reproduced damage progression caused by thermal cycling and revealed several key findings.

First, under the fixed displacement condition, thermal stress arises due to the restriction of thermal deformation, leading to significant stress relaxation, energy dissipation, entropy generation, and ultimately material failure. For example, in the case of the 300 to 400 K temperature range, failure occurred after approximately 15 s, corresponding to about 7.5 thermal cycles. When the temperature fluctuation range was reduced to 340 to 400 K, failure occurred at the eighteenth cycle. Second, even under the free expansion condition—where thermal deformation is allowed—temperature gradients within the material result in internal stresses and localized damage. The results also clarified that larger temperature fluctuations lead to faster damage accumulation, even when the average temperature is comparable. These observations suggest that the width of temperature variation plays a more crucial role than the average temperature in determining thermal fatigue behavior.

Additionally, this study demonstrated that damage accumulation can be predicted based on viscoelastic dissipation and the time–temperature superposition principle, without requiring extensive physical testing. While the current model treats the polymer as a homogeneous material, it provides a solid foundation for future development, including the separate treatment of fibers, resin, and interfaces in composite materials. Such extensions are expected to enable the application of this approach to more realistic composite structures and contribute to the improvement of product durability and design reliability in thermal environments.

Overall, this work offers a novel and practical numerical methodology for simulating thermal fatigue in polymers, which is a critical step toward understanding and managing thermal damage in high-performance engineering applications.

## Figures and Tables

**Table 1:** Material properties of the Maxwell elements, thermal parameters, entropy criterion, and damage variables used in the simulation.

Maxwell Elements			Elasticity		Damage Variables	
i	$E_{i}$ [MPa]	$\eta_{i}$ [MPa∙s]	$E_{0}$ [MPa]	4260	Activation energy ΔH [kJ]	100
1	284	4.5 × 10^2^	ν	0.33	$\mathrm{Reference}\;\mathrm{temperature}\;T_{R}$ [K]	300
2	284	3.3 × 10^3^			$\mathrm{Damage}\;\mathrm{parameter}\;\alpha_{d}$	4
3	284	1.2 × 10^5^	**Nonlinearity**		$\mathrm{Damage}\;\mathrm{criterion}\;D_{cr}$	0.25
4	284	1.9 × 10^6^	$\sigma_{0}$ [MPa]	70	$\mathrm{Entropy}\;\mathrm{criterion}\;S_{cr}$ [kJ]	0.4
5	284	1.8 × 10^7^	α	2		
6	284	1.4 × 10^8^	m	7		
7	284	8.5 × 10^8^				
8	284	5.0 × 10^9^	**Thermal Parameters**			
9	284	3.0 × 10^10^	Specific heat c [J/kg∙K]	1130		
10	284	1.9 × 10^11^	Density ρ [kg/m^3^]	0.0014		
11	284	1.4 × 10^16^	Heat transfer coefficient h [W/m^2^·K]	1		
12	284	1.3 × 10^19^	Thermal conductivity λ [W/m·K]	0.3		
13	284	2.1 × 10^22^	Thermal expansion coefficient [/K]	4 × 10^−5^		
14	284	1.3 × 10^26^				
15	284	2.5 × 10^29^				

## Data Availability

The raw data supporting the conclusions of this article will be made available by the authors on request.
